# A passive electronic medical record‐based cognitive risk calculator predicts neuropsychological performance in older adults

**DOI:** 10.1002/alz.089480

**Published:** 2025-01-09

**Authors:** Saket A Saxena, Olivia Hogue, Michael B Rothberg, Anita D Misra‐Hebert, Alexander P Milinovich, Elizabeth R Pfoh, Robyn M Busch, Kamini Krishnan, Robert J Fox, Michael W Kattan, Darlene Floden

**Affiliations:** ^1^ Cleveland Clinic, Cleveland, OH USA

## Abstract

**Background:**

Stratifying risk of cognitive decline for an individual patient can be difficult in primary care settings where advanced biomarkers are usually not available. Passive risk calculators that capitalize on existing information contained in the electronic medical record (EMR) hold promise, but most are developed using EMR documentation of cognitive decline which is highly unreliable. This prospective study used objective cognitive testing to build a multivariable cognitive risk model based on EMR records.

**Methods:**

338 primary care patients aged 60 and older who had at least two visits over the previous 5 years completed brief neuropsychological testing to establish current cognitive function and consented to access to their medical records. Records from the preceding 5 years were mined for health status indicators (e.g., BMI, pulse, blood pressure), diagnoses, medications, demographic, and socioeconomic information and entered into a multivariable logistic regression model using Harrell’s step‐down selection. To account for changes in variable values from visit to visit, binary variables (e.g. medications, diagnoses, smoking status) were classified as absent or ever‐present during patient follow‐up, while first, last, and “worst” values were retained for continuous variables (e.g. blood pressure, pulse). Bootstrap resampling was used for internal validation. Model was adjusted for optimism and fit evaluated using calibration curves.

**Results:**

The sample was 59% female and 80% white, with a mean age of 72.5 (SD 7.0) and 15.5 years of education (SD 2.6)). Eighty patients (24%) were cognitively impaired. The model that best identified impairment included age, most recent pulse, race, presence of mood disorder diagnosis, family history of neurological diagnosis, NSAID use, and most recent systolic blood pressure. After adjusting for optimism, the discrimination index was 0.71, indicating good discrimination. Results from the model were transformed to yield patient‐specific probability scores used to trigger risk flag in the patient’s medical record.

**Conclusions:**

An EMR‐based risk calculator successfully discriminated between patients with and without cognitive impairment. Strategies for implementing the cognitive risk flag into clinical practice are discussed.

